# Asymmetric Dimethylarginine Is a Marker of Endothelial Dysfunction in Thrombotic Antiphospholipid Syndrome Patients

**DOI:** 10.3390/ijms232012309

**Published:** 2022-10-14

**Authors:** Natasa Stanisavljevic, Ljudmila Stojanovich, Aleksandra Djokovic, Brankica Todic, Violeta Dopsaj, Jovica Saponjski, Dusan Saponjski, Olivera Markovic, Cristina Belizna, Marija Zdravkovic, Dragomir Marisavljevic

**Affiliations:** 1 University Clinical Center “Bezanijska kosa”, Bezanijska kosa bb, 11080 Belgrade, Serbia; 2 Medical Faculty, University of Belgrade, 11000 Belgrade, Serbia; 3 Special Hospital “Zutic”, 11000 Belgrade, Serbia; 4 Faculty of Pharmacy, University of Belgrade, 11000 Belgrade, Serbia; 5 Clinic of Cardiology, University Clinical Center of Serbia, 11000 Belgrade, Serbia; 6 Center of Radiology and MR, University Clinical Center of Serbia, 11000 Belgrade, Serbia; 7 Internal Medicine Department Clinique de l’Anjou, Angers, Vascular and Coagulation Department, University Hospital Angers, 49100 Angers, France

**Keywords:** antiphospholipid syndrome, lupus erythematosus, systemic, inflammation

## Abstract

Objective: The potential contribution of asymmetric dimethylarginine (ADMA) and high-sensitivity C reactive protein (hsCRP) to endothelial dysfunction in APS patients has not been studied in detail, until now. The study involved 105 APS patients (59 diagnosed with primary APS (PAPS) and 46 APS associated with systemic lupus erythematosus (SAPS)) who were compared to 40 controls. Endothelial dysfunction was assessed by measurement of flow-mediated dilatation (FMD) and glyceryl trinitrate dilatation (NMD) of the brachial artery. ADMA (micromol/L) was analyzed by ELISA. Results: FMD in patients with APS was significantly lower than that of the controls (*p* < 0.001), with no difference between the PAPS and the SAPS groups. ADMA and hsCRP concentrations were significantly higher in the patient cohort than in the control group (*p* < 0.001, *p* = 0.006, respectively), as was the case with the SAPS group as compared to the PAPS group (*p* < 0.001, *p* = 0.022, respectively). FMD impairment correlated to ADMA (ρ 0.472, *p* < 0.001) and to hsCRP (ρ 0.181, *p* = 0.033). In the regression model, the ADMA concentration confirmed the strength of its association (B 0.518, SE 0.183, Wald 8.041, *p* = 0.005, Exp(B) 1.679, 95% CI 1.174–2.402) to FMD impairment. The synergistic probability model of ADMA and hsCRP caused FMD impairment when the positivity of β2GPIIgG was added. ADMA may be used as a simple and low-cost tool for verifying the presence of endothelial dysfunction in APS patients. According to the results of the study, we could presume that hsCRP, together with aPL, has a preparatory effect on the endothelium in causing endothelial dysfunction.

## 1. Introduction

Antiphospholipid syndrome (APS) is a potentially lethal disease with a 10-year survival probability of about 90%, with fatal thrombosis as the most common factor in mortality [1]. APS can manifest without any other underlying disorder (primary APS—PAPS) or accompanied by another autoimmune disease, most commonly systemic lupus erythematosus (APS associated with systemic lupus erythematosus—SAPS). The diagnosis of APS is based on the clinical criterion of a thrombotic/pregnancy complication together with repeated positivity for one or more antiphospholipid antibody (aPL) in a medium or high titer [2].

Pathological endothelial activation seems to initiate the prothrombotic state in APS. Direct proof that endothelial dysfunction in APS patients is based on impairment of nitric oxide (NO) production is that plasma antiphospholipid antibody (aPL) levels are inversely correlated with urinary NO metabolite excretion, and APS patients have lower levels of plasma nitrites compared to control subjects [3,4]. The endogenous inhibitor of the endothelial isoform of NO synthetase (eNOS)-asymmetric dimethyl arginine (ADMA) is responsible for endothelial vasodilator dysfunction in individuals with coronary and peripheral arterial disease, renal impairment, as well as in those with risk factors, such as hypercholesterolemia, hypertension, insulin resistance, and aging [5]. Elevated ADMA levels have been found in patients affected by systemic autoimmune disorders, for example, RA [6] and SLE [7]. High-sensitivity C reactive protein (hs-CRP) has been identified by multiple guidelines as a biomarker of the risk factor for atherosclerotic cardiovascular disease [8,9]. However, another effect of CRP is also of interest. It downregulates eNOS transcription in endothelial cells and destabilizes eNOS mRNA with a resultant decrease in both basal and stimulated NO release [10].

The objective of this study is to evaluate the potential contribution of ADMA and hsCRP to endothelial dysfunction in APS patients, in a clinical setting.

## 2. Results

### 2.1. Clinical and Laboratory Characteristics

There was no significant difference between the groups regarding traditional cardiovascular risk factors (age, BMI, hypertension, diabetes, smoking habit, triglycerides, total cholesterol, HDL, and LDL) (Table 1). Mean disease duration in PAPS patients was 4.41 ± 2.9 years, and in SAPS patients, 5.52 ± 2.95 years.

Thrombosis was diagnosed in 68 (64.8%) PAPS and SAPS patients. Arterial thrombosis was recorded in 42 (40.4%), and venous thrombosis in 40 (38.5%) patients, since some patients had several thrombotic events (simultaneously or consecutively). The arterial system was affected in 15 (25.9%) PAPS and 27 (58.7%) SAPS patients (*p* = 0.001). Vascular events related to the venous system were present in 22 (37.9%) PAPS and 18 (39.1%) SAPS patients (*p* = 0.901). Fetal losses were present in 46 (79.3%) PAPS and 24 (61.5%) SAPS patients (*p* = 0.056).

The distribution of aPL in the PAPS and SAPS groups revealed a statistically significant difference in the presence of aCL IgG and β2GPI IgG antibodies, as presented in Table 2. More than one type of antibody (category I) was present in 34 (57.6%) PAPS patients and 38 (82.6%) SAPS patients (*p* = 0.006). Triple aPL positivity was present more often in the SAPS group (20.3% vs. 39.1, respectively, *p* = 0.034) while there was no statistically significant difference between the other category groups (Table 2).

There was no significant difference when analyzing aPL antibody categories and thrombotic events, but triple positivity was significant for overall thrombosis (*p* = 0.039) and venous events (*p* = 0.015).

### 2.2. Brachial Artery Measurements

The basal artery diameter in PAPS was 3.73 ± 0.56 mm; in SAPS, 3.71 ± 0.72 mm; and in the controls, 3.59 ± 0.71 mm. There was no significant difference between the groups with respect to the basal brachial artery diameter (*p* = 0.336).

It was found that the percentage of FMD dilatation in patients with PAPS and SAPS (9.88 ± 4.36 (95% CI 8.24 to 11.06) and 11.27 ± 6.3 (95% CI 8.39 to 12.49), respectively, *p* = 0.834) was significantly lower than that of the control group (15.12 ± 5.6 (95% CI 14.36 to 18.67); *p* < 0.001). The difference in the percentage of NMD response was similar in the groups of APS patients (PAPS 15.7 ± 8.33; SAPS 14.59 ± 7.85; *p* = 0.298) and controls (15.81 ± 6.23, *p* = 0.407). Impairment of FMD (FMD <10% basal value) was found in 30 (28.6%) APS patients and 5 (12.5%) controls (*p* = 0.043).

The percentage of FMD relative to basal diameter correlated to age (ρ −0.251, *p* = 0.002), hypertension (ρ −0.181, *p* = 0.031), and almost all lipid fractions: cholesterol (ρ −0.202, *p* = 0.019), triglycerides (ρ −0.252, *p* = 0.003), and LDL (ρ −0.191, *p* = 0.027). However, in regression analysis, only age showed to be an independent variable for the percentage of FMD dilatation (B 0.076, Wald 10.014, *p* = 0.002, Exp(B) 1.079, 95% CI 1.029–1.131). FMD impairment correlated to a thrombotic event, which was a clinical manifestation of APS (ρ 0.282, *p* = 0.029), and there was no correlation to aPL.

### 2.3. Analysis of ADMA and hsCRP

The significant differences in ADMA and hsCRP concentrations between groups are shown in Table 3.

**Figure 1:**
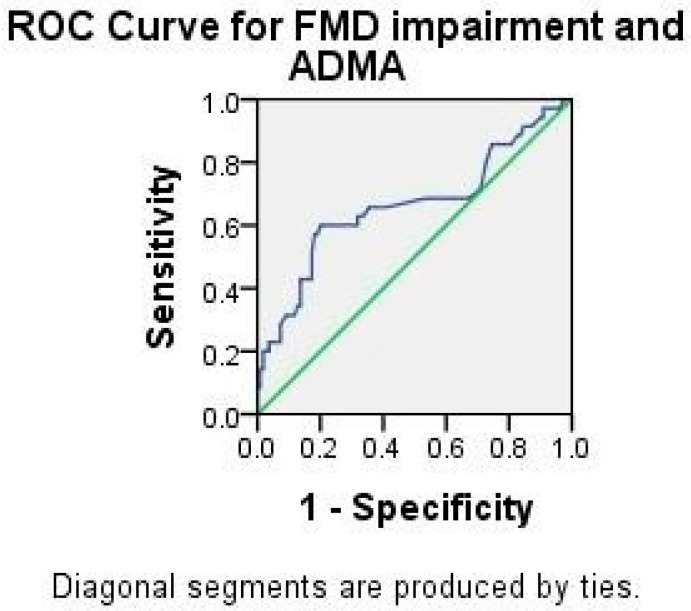
ROC analysis for ADMA and FMD impairment.

ADMA correlated to both the percentage of FMD dilatation and FMD impairment (both *p* < 0.001), but not to NMD. Figure 1 shows the ROC curve for ADMA and FMD impairment (AUC 0.663, *p* = 0.004, 95% CI 0.547–0.779). It is interesting that values of ADMA ranging from 0.715 to 0.925 have the same sensitivity of 60%, but there is a rise in specificity ranging from 68% to 80%.

In the control group, no significant correlations between ADMA concentration and risk factors (BMI, hypertension, diabetes, smoking habit, or lipid fractions) were found. In the PAPS group, ADMA concentration correlated to age (ρ 0.376, *p* = 0.003), presence of hypertension (ρ 0.485, *p* = 0.001), and smoking habit (ρ 0.297, *p* = 0.024). In the SAPS group, ADMA correlated to SLEDAI (ρ 0.480, *p* = 0.001), a high titer of B2IgG (ρ 0.193, *p* = 0.049), and to any positivity of aCL IgG (ρ 0.208, *p* = 0.033).

**Figure 2:**
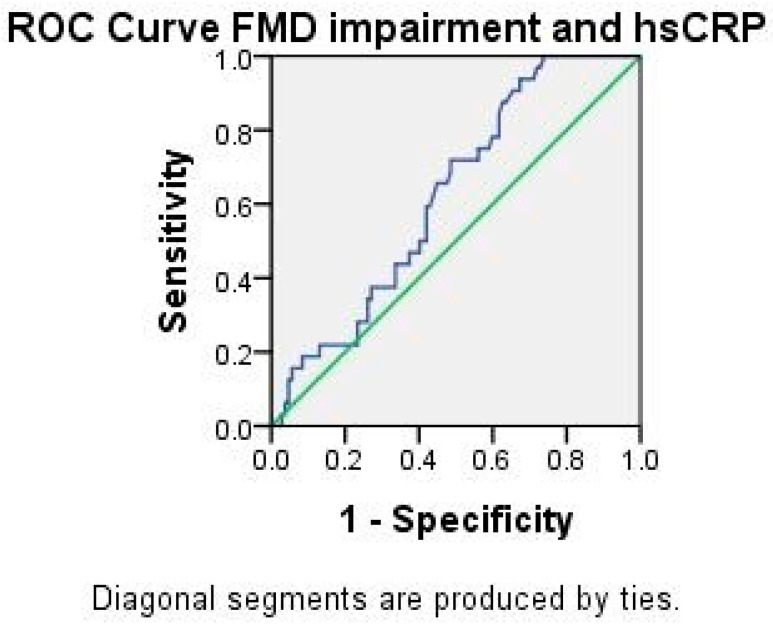
ROC analysis for hsCRP and FMD impairment.

The concentration of hsCRP correlated to FMD impairment (ρ 0.181, *p* = 0.033). Figure 2 shows the ROC curve for hsCRP and FMD impairment (AUC 0.624, *p* = 0.033, 95% CI 0.526–0.722). The discriminative hsCRP cut off value of 0.96 had a sensitivity of 72% and a specificity of 51% for FMD impairment. The concentration of hsCRP in the control group showed strong correlations to lipid fractions: cholesterol (ρ 457, *p* = 0.008), triglycerides (ρ 0.685, *p* = 0.001), HDL (ρ −0.695, *p* = 0.001), and LDL (ρ 0.619, *p* = 0.001), and a weaker correlation to hypertension (ρ 0.390, *p* = 0.016) and smoking habit (ρ 0.430, *p* = 0.007). In the APS group, hsCRP correlated to a medium/high titer of B2GPI IgG (ρ 0.225, *p* = 0.024) and triple aPL positivity (ρ 0.233, *p* = 0.019), but there was no significant correlation to the clinical manifestation of APS.

**Figure 3:**
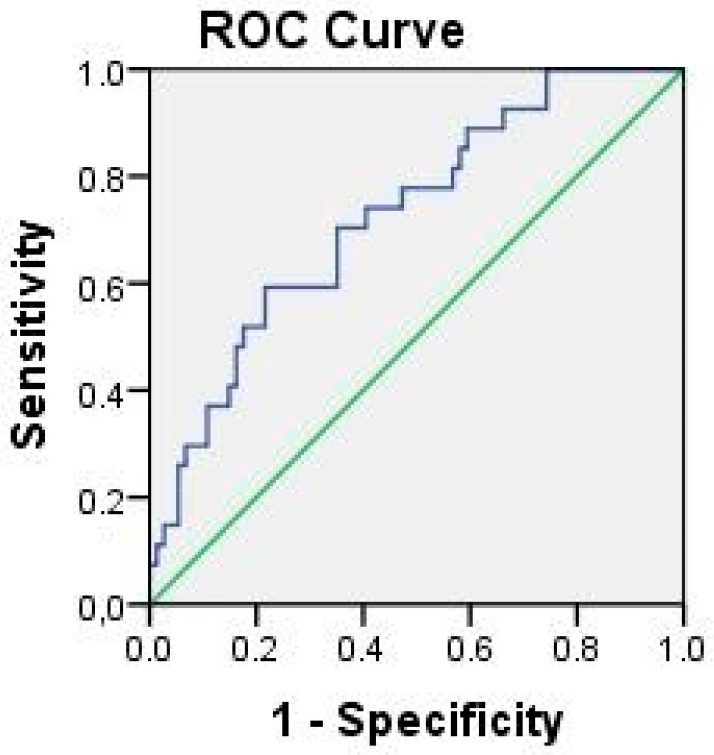
ROC curve for FMD impairment—predicted probability of synergistic effect of B2GPIgG, hsCRP, and ADMA.

The concentrations of hsCRP and ADMA showed a positive correlation to each other (ρ 0.190, *p* = 0.025). Since each of them had an impact on endothelial dysfunction in APS patients, we tested the hypothesis in order to determine whether the effect of these variables on endothelial dysfunction in APS patients was synergistic or separate. In binary logistic analysis, ADMA concentration confirmed the strength of its association with FMD impairment (B 0.518, SE 0.183, Wald 8.041, *p* = 0.005, Exp(B) 1.679, 95% CI 1.174–2.402) in contrast to the hsCRP concentration, which was not statistically significant. Next, we analyzed the effect of ADMA and hsCRP and their synergistic probability to cause endothelial dysfunction on the ROC curve. The ROC curve failed to show any difference, but proved to be rather similar to the ADMA ROC curve (AUC 0.638, *p* = 0.018, 95% CI 0.515–0.760) and we concluded that higher ADMA concentrations contributed to endothelial dysfunction in APS patients more than hsCRP concentrations did. Due to the fact that B2GPI IgG correlates to both ADMA and hsCRP, we tested the model of predicted probability for endothelial dysfunction of all three variables (B2GPI IgG, ADMA and hsCRP) as one. The results showed that this variable was the best predictor of FMD impairment in APS patients (Figure 3; (AUC 0.726, *p* = 0.001, 95% CI 0.618–0.834)).

## 3. Discussion

ADMA and hsCRP are acute-phase plasma proteins induced by different mechanisms. ADMA rises through activation of inducible eNOS, and CRP is regulated by pro-inflammatory cytokines, though non-acute phase elevations may occur in several chronic inflammatory diseases, such as atherosclerosis and autoimmune diseases such as RA and SLE [7]. The current study reveals that a significant number of APS patients (PAPS and SAPS) display a low-grade inflammatory state that is presumed to be due to the disease itself, when endothelial dysfunction is present. In this study, ADMA has proven to be a relevant biomarker of endothelial dysfunction, without a synergistic effect, but rather a separate effect of hsCRP on the endothelium, potentiated by aPL.

Cohorts of APS patients have shown similar clinical and laboratory profiles, either among PAPS patients or patients with APS associated to SLE (SAPS) [1,11]. Some studies found a higher prevalence of β2GPI IgG antibodies in PAPS compared to SLE-associated APS [12,13]. It has been found that LA is the strongest individual risk factor for venous thrombosis, as are triple and double antibody positivity when multiple factors are taken into consideration [14,15,16]. We have found higher rates of arterial thrombosis, as well as a higher prevalence of aCL IgG and β2GPI IgG antibodies, and a higher rate of double and triple positivity in the SAPS patient group. In addition, triple positivity was confirmed as a high-risk profile for a thrombotic event.

The measurement of flow mediated vasodilatation (FMD) is a non-invasive, inexpensive, reproducible method for endothelial dysfunction assessment, reflecting the ability of endothelial cells to induce NO-mediated vasodilatation as a response to blood flow provoked vessel wall shear stress [17]. Since previous FMD studies have reported differences regarding their adherence to key methodological issues, we have overcome this problem by means of a well-defined analysis protocol [18]. FMD is reduced in subjects with atherosclerosis and cardiovascular risk factors—smokers [19], subjects with high cholesterol levels [20], arterial hypertension [21], diabetes [22], obesity, aging [23], and renal failure [24]. Endothelial dysfunction measured by the FMD of the brachial artery has already been proven in patients with PAPS and SLE [25,26]. Much lower FMD in PAPS patients is associated to aCL positivity, and lower FMD is more commonly found in arterial compared to venous involvement in these patients [4,27]. In patients with SLE, early endothelial dysfunction is present in those subjects who have low FMD, which correlates with modified or native LDL, as well as soluble E-selectin and ICAM-1 serum levels [25,28]. The present study confirmed that there is a reduction in the percentage of brachial artery dilatation in response to shear stress, similarly to a majority of the aforementioned studies. This dilatation percentage was even lower in SAPS than in PAPS patients. Although lipid fractions and hypertension correlated to FMD impairment in APS patients, age (but not disease duration) proved to be the most important risk factor. FMD impairment likely correlates to thrombosis as the initial step before its clinical manifestation in APS patients. Aging is a known risk factor for venous thromboembolism in the general population, but after the age of 50, the risk increases exponentially [29]. Our APS patients were mostly younger than 50 years (mean 42 years), but, obviously, with a lower threshold. It could be presumed that some predetermined factors affect the initial features of the APS patients’ vessel walls, causing endothelial dysfunction, or that these factors are a feature of accelerated aging in these patients, and not a consequence of disease duration.

A healthy endothelium supports vascular homeostasis and acts as an antithrombotic surface. The key endothelium-derived relaxing factor is nitric oxide (NO). The role of NO is also the inhibition of platelet adhesion and aggregation, leukocyte adhesion and migration, and smooth muscle proliferation. Therefore, the impairment of NO production leads towards a prothrombotic phenotype [30]. In PAPS patients, aCL antibody titers inversely correlate with urinary NO levels. This is in contrast with the results found in SAPS patients [4]. In addition, the basis for human studies relies on the fact that monomeric B2GPI has no effect on endothelial NO synthetase (eNOS) activation, whereas dimerized B2GPI completely inhibits eNOS [31]. Elevated levels of ADMA (endogenous inhibitor of eNOS) have been found in patients affected by systemic autoimmune disorders, for example, RA [6] and SLE [7,32].

There are limited data on ADMA levels in APS patients. Only one study by Mayer-Pickel et al. has shown increased concentrations of ADMA in pregnancies with APS, compared to normal pregnancies [33]. In our study, a higher concentration of ADMA was found in patients with APS than in controls, and it was also higher in SAPS as compared to PAPS patients. This implies that the endogenous inhibitor of eNOS (ADMA) is responsible for lower production of NO in APS patients. ADMA correlated to FMD impairment, and it is interesting that the values of ADMA ranging from 0.715 to 0.925 had the same sensitivity of 60% (true positive), but that the specificity rose from 68% to 80% (true negative) on the ROC curve, highlighting this value range as a reliable variable. Since the value of 0.71 was the mean value in our group of PAPS patients, it could be assumed that ADMA is a relevant biomarker of endothelial dysfunction in APS patients. On the other hand, the following question arises: is ADMA production higher in patients with APS or is this the consequence of an APS pathognomonic feature, such as aPL activity? Although other studies have demonstrated that lipid fractions, hypertension, aging, and other factors affect ADMA concentration [5], our results do not show these relations. Such a difference is probably due to the well-balanced traditional cardiovascular risk factors in our groups. We did not find a correlation between ADMA and the clinical manifestation of APS. A positive correlation of ADMA concentration to SLEDAI in SLE patients is already known of [7,32]; however, our SAPS patients had a very low SLEDAI score (stable disease).

High-sensitivity C reactive protein (hs-CRP) is more precise than standard CRP when measuring baseline (i.e., normal) concentrations, and enables measurement of chronic inflammation. Measurement of hsCRP in APS is unreliable when distinguishing PAPS from SAPS [34]. Although hs-CRP serum levels were significantly higher in patients with systemic lupus erythematosus as compared to healthy controls, it seems that this marker is not a good indicator for disease activity [35]. On the other hand, low-grade inflammation and immune activation occur in thrombotic PAPS, and relate to clinical features and aPL levels [36].

In our study, the hsCRP concentration was higher in the APS (more SAPS than PAPS) than in the control group, confirming APS to be a “low grade” inflammatory state. The link between hsCRP and hyperlipidemia has not proven to be strong enough, since there was no correlation of lipid fractions to the hsCRP concentration in the SAPS group (where hsCRP concentration was the highest). However, it could be that SLE patients with an APS diagnosis take care of their diet and lifestyle more than PAPS patients do.

The concentration of hsCRP and ADMA showed a direct positive correlation, but only ADMA concentration confirmed the strength of its association to FMD impairment. The effect of hsCRP on endothelial dysfunction was separate and not synergistic with the ADMA effect. The proposed mechanism of decreased eNOS mRNA synthesis caused by CRP, resulting in endothelial dysfunction, is not the only one. Autoantibodies from patients with APS infused into mice do not promote thrombus formation in the absence of vessel wall injury [37]. Our statistical model of predicted probability for endothelial dysfunction of all three variables (B2GPI IgG, ADMA, and hsCRP) as one confirmed the assumption that hsCRP is a preparatory factor affecting the vascular endothelium and causing the full aPL effect. The study is limited by the rather the small number of participants, and the conclusion still needs to be further confirmed by a larger sample size and prospective cohort studies.

## 4. Materials and Methods

### 4.1. Patients

This cross-sectional case–control clinical study included a total of 145 Caucasian patients. This group consisted of 105 thrombotic APS patients: 59 PAPS (58 female and 1 male, mean age 41 ± 11.02 years, range 22–68 years) and 46 SAPS (39 female and 7 male, mean age 41.76 ± 12.39 years, range 17–67 years), compared to 40 healthy aPL negative individuals without a history of thrombotic events, recruited as the control group (34 female and 6 male, mean age 44 ± 10.18 years, range 23–78 years). All patients met the revised Sydney criteria for APS [2] and the American College of Rheumatology (ACR) classification criteria [38] for SLE. Disease activity was assessed at the time of enrolment using the Systemic Lupus Erythematosus Disease Activity Index (SLEDAI) [39]. Clinical data and medication were retrieved from the clinical database and patient records. Patients with APS were included consecutively from the year 2013 to the year 2016 in a prospective manner at the University Hospital Medical Center “Bežanijska kosa.” Exclusion criteria included conditions that could influence endothelial perturbation (acute or chronic infection, malignancy, or marked renal and liver impairment). SLE patients were without active CNS lupus manifestations, lupus nephritis, cytopenias, or any skin manifestations. The mean SLEDAI for SAPS patients ranged: 4.87 ± 2.75. The patients were treated according to current guidelines [40].

The study was approved by the local ethical committee and written informed consent was obtained from all individual participants included in the study. All procedures conducted on patients were in accordance with the Helsinki Declaration and its later amendments or comparable ethical standards.

### 4.2. Methods

After overnight fasting (12 h) and after 24 h without intensive physical activity, patients rested prior to venipuncture. The blood samples were collected (tourniquet use was never longer than 3 min), centrifuged for 10 min at 3000 rpm, and stored at −70 °C no longer than one month before analysis, in keeping with the manufacturer’s instructions.

LA screening was based on the use of two different screening tests: the tissue thromboplastin inhibition test and diluted activated partial thromboplastin time with phospholipid neutralization, according to ISHT recommendations [41]. Anticardiolipin (aCL: IgG/IgM) and anti-β2glycoprotein I (β2GPI: IgG/IgM) antibodies were measured by an enzyme-linked immunosorbent assay (ELISA, Binding Site) and expressed in G phospholipid (GPL) or M phospholipid (MPL) units (GPL-U and MPL-U). The ranges for positive levels were defined as low (11–40 U/mL), medium (41–99 U/mL), and high (>100 U/mL). According to the type of positive aPL, patients with APS were classified into the following categories: category I, where two or more laboratory criteria are present in combination; category IIa, where the lupus anticoagulant (LA) is present alone; category IIb, where anticardiolipin antibodies (aCL) are present alone; and category IIc, where anti-β2 glycoprotein-I antibodies (anti β2GPI) are present alone [42]. Antinuclear antibodies were determined by indirect immunofluorescence on mouse livers and on the HEp-2 cell substrate. Anti-double stranded DNA (anti-dsDNA) antibodies were determined by use of the ELISA method (Binding Site).

High-sensitivity CRP was analyzed by applying the immunoturbidymetric method (Cobas 511c, Roche, Rotkreuz, Switzerland). ADMA was determined by the ELISA method (Cusabio kit). The lipid status, as total cholesterol, high-density lipoprotein (HDL) cholesterol, low-density lipoprotein (LDL) cholesterol, and triglycerides, was assessed employing the routine methods (Olympus System kit, Olympus AU 2700, Hamburg, Germany).

All patients and controls underwent evaluation of endothelial function by means of a non-invasive assessment of endothelium dependent flow mediated dilatation (FMD) and endothelium independent, nitroglycerine-mediated vasodilatation (NMD) of the brachial artery by B mode ultrasonography, using a standardized procedure [17]. Our group employed this method in the previous study, where it has been described in detail [18].

### 4.3. Statistical Analysis

Descriptive statistics were used to summarize clinical characteristics of the study group. The Shapiro–Wilk test (*p* > 0.05, normal distribution of data assumed) and Q-Q plots were used to confirm the assumption of normal distribution. Numerical variables were expressed as the mean ± SD in case of normal distribution, median (interquartile range) if a variable did not follow normal distribution, and as a percentage for categorical data. If normal distribution was met, the one-way ANOVA with the Tukey correction was used for group comparisons; otherwise, the non-parametric Kruskal–Wallis test was applied. The Chi-squared test was used to compare categorical variables. The correlation between two quantitative variables was determined by Spearman’s correlation test. ROC analysis was used to determine AUC parameters and cut off values. A *p*-value below 0.05 was considered significant. All analyses were performed by means of the SPSS statistical analysis software, Version 20.0 (SPSS, Chicago, IL, USA).

## 5. Conclusions

Loss of endothelial function and subsequent thrombosis are hallmarks of APS. The exact role of aPL is still to be elucidated due to the lack of standardization, natural variability of its concentrations, and the number of possible “active” aPLs. We have shown that ADMA concentration is a reliable marker for endothelial dysfunction in APS patients. In addition, it could be assumed that hsCRP (characterizing APS as a low inflammatory state) acts on the endothelial surface, preparing it for the full aPL effect. Determining the concentration of ADMA and CRP in common clinical practice could indicate a possible, more aggressive manifestation of the APS clinical picture, and emphasize the impact of endothelial dysfunction.

## Figures and Tables

**Table 1:** Risk factors in APS patients and healthy control group parameters are presented as mean ± SD.

Variables	PAPS N = 90	SAPS N = 50	Control Group N = 40	*p*
BMI	24.73 ± 4.96	25.44 ± 4.23	25.14 ± 5,42	0.424
Hypertension, n (%)	23 (38.9%)	14 (30%)	11 (27.5%)	0.473
Diabetes, n (%)	2 (3.4%)	2 (4.3%)	1 (2,5%)	0.902
Smoking, n (%)	17 (28.8%)	9 (30.4%)	7 (17.5%)	0.902
Triglycerides, (mmol/L)	1.28 ± 1.12	1.24 ± 0.55	1.26 ± 0.58	0.426
Total cholesterol, (mmol/L)	5.39 ± 1.20	5.28 ± 1.0	5.46 ± 0.91	0.733
HDL-C, (mmol/L)	1.52 ± 0.48	1.45 ± 0.36	1.51 ± 0.41	0.921
LDL-C, (mmol/L)	3.32 ± 1.12	3.25 ± 0.88	3.37 ± 0.88	0.567

Body mass index, BMI; high density lipoprotein, HDL; low density lipoprotein, LDL.

**Table 2:** The distribution of aPL in the PAPS and SAPS groups.

	PAPS N (%)	SAPS N (%)	*p* Value
aCL IgM median—U/mL; IQR	29 (49.2%) 12.1 (26.8)	31 (67.4%) 24.6 (42.4)	0.051 0.068 *
aCL IgG median—U/mL; IQR	25 (42.4%) 8.9 (26.4)	31 (67.4%) 22.6 (36.5)	**0.011** **0.014** *
ß2GPI IgM median—U/mL; IQR	23 (39%) 5.7 (16.7)	23 (50%) 11.1 (41.2)	0.259 0.130 *
ß2GPI IgG median—U/mL; IQR	18 (30.5%) 5.7 (18.5)	26 (56.5%) 17.8 (37.5)	**0.007** **0.031** *
LA	24 (40.7%)	24 (52.2%)	0.241
Category IIa	6 (10.2%)	2 (4.3%)	0.265
Category IIb	12 (20.3%)	5 (10.9%)	0.191
Category IIc	7 (11.9%)	3 (6.5%)	0.355
Category I	34 (57.6%)	38 (82.6%)	**0.006**
Triple positivity	12 (20.3%)	18 (39.1%)	**0.034**

aPL—antiphospholipid antibody; IQR—interquartile range; *p* value—X^2^ test; * *p* value—Mann–Whitney.

**Table 3:** Concentrations of ADMA and hsCRP.

Parameter	PAPS	SAPS	Control	*p* *	*p* **
Mean ADMA μmol/L (min–max)	0.71 (0.35–1.38)	1.96 (0.55–5.81)	0.56 (0.22–0.97)	**<0.001**	**<0.001**
Mean hsCRP mg/L (min–max)	2.98 (0.05–53.9)	7.94 (0.16–62.6)	1.61 (0.05–9.44)	**0.006**	**0.022**

*p* *—comparison between PAPS and SAPS; *p* **—comparison between APS and control.

## Data Availability

All data relevant to the study are included in the article or uploaded as supplementary information. De-identified participant data may be available upon reasonable request by sending an email to nacastanisavljevic@gmail.com.
